# Colloidal lignin particles for Pickering emulsion stabilization: multifunctional properties and prospects for cosmetic applications

**DOI:** 10.1007/s13659-026-00594-3

**Published:** 2026-03-03

**Authors:** Giovana Colucci, Alírio Egídio Rodrigues, Maria Filomena Barreiro

**Affiliations:** 1 CIMO, LA SusTEC, Instituto Politécnico de Bragança, Campus de Santa Apolónia, 5300-253 Bragança, Portugal; 2 LSRE-LCM, ALiCE, Faculty of Engineering, University of Porto, Rua Dr. Roberto Frias, 4200-465 Porto, Portugal

**Keywords:** Lignin, Colloidal lignin particles, Pickering emulsions, Pickering stabilizers, Cosmetics

## Abstract

Lignin, the most abundant aromatic biopolymer on Earth, is abundantly found as a by-product of biomass processing industries. Lignin presents remarkable properties, including antioxidant, UV-blocking, thermal stability, and antimicrobial; thus being considered a valuable feedstock to replace synthetic and fossil-based resources. Colloidal lignin particles (CLPs) are a promising strategy to overcome technical barriers in lignin valorization and have attracted increasing interest to be applied as multifunctional ingredients into several fields, including agriculture, biomedical, construction, and more recently, cosmetics. For this latter, CLPs stand out as promising Pickering stabilizers, creating opportunities for the development of surfactant-free cosmetic formulations. This review provides a current state of lignin research, focusing on the production of CLPs and Pickering emulsions and the key factors influencing their formation and stability. Recent progress in CLPs-stabilized Pickering emulsions is thoroughly addressed. Furthermore, it highlights advances on the multifunctional attributes of CLPs, including antioxidant activity, UV shielding, and demonstrated safety for cosmetic applications. The review concludes by discussing current challenges and future research directions for advancing the use of CLPs as sustainable materials in cosmetic science.

**Graphical Abstract:**

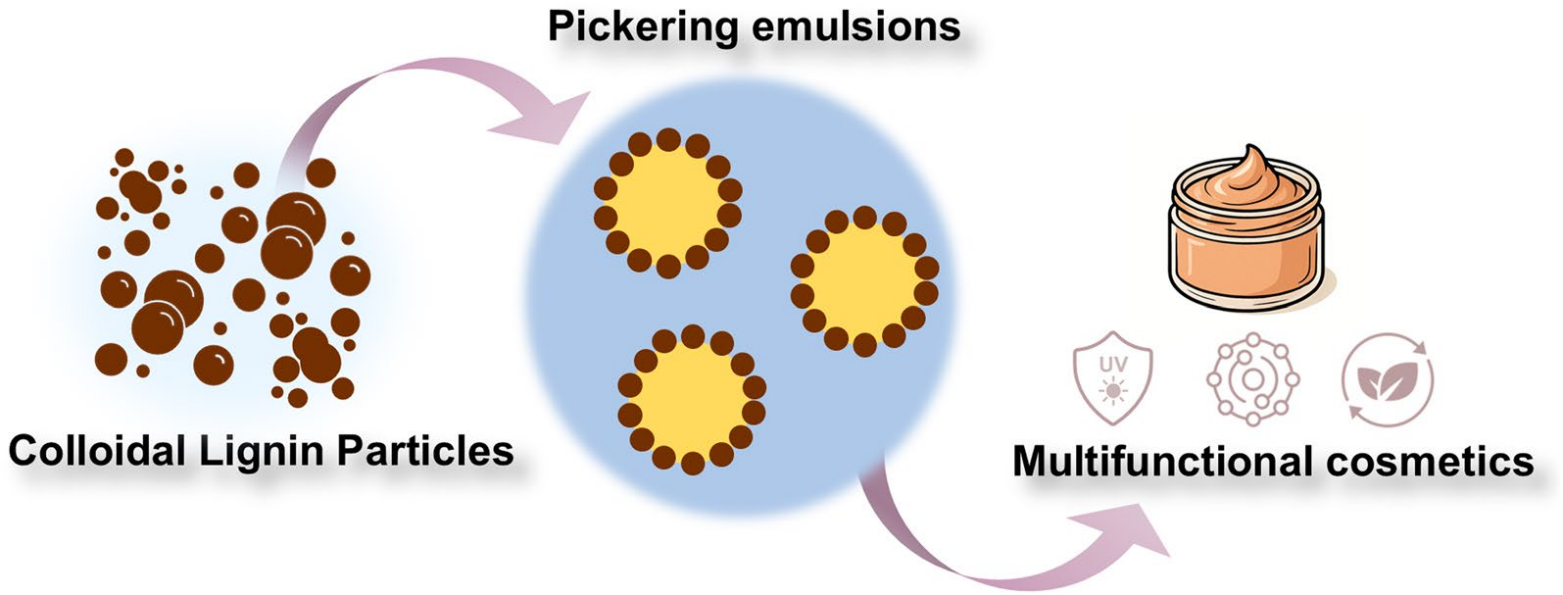

## Overview of lignin chemical structure

Lignin is a heterogeneous phenolic macromolecule found in all vascular plants and represents the most abundant aromatic resource in nature [1, 2]. It is embedded within the plant cell wall matrix, in combination with cellulose and hemicellulose, and its concentration varies across plant types. Lignin plays a vital role in plant growth and development by imparting rigidity and strength to cell walls, controlling water transport, and protecting against environmental stresses such as ultraviolet radiation and attacks by microorganisms and insects [3, 4].

**Fig. 1:**
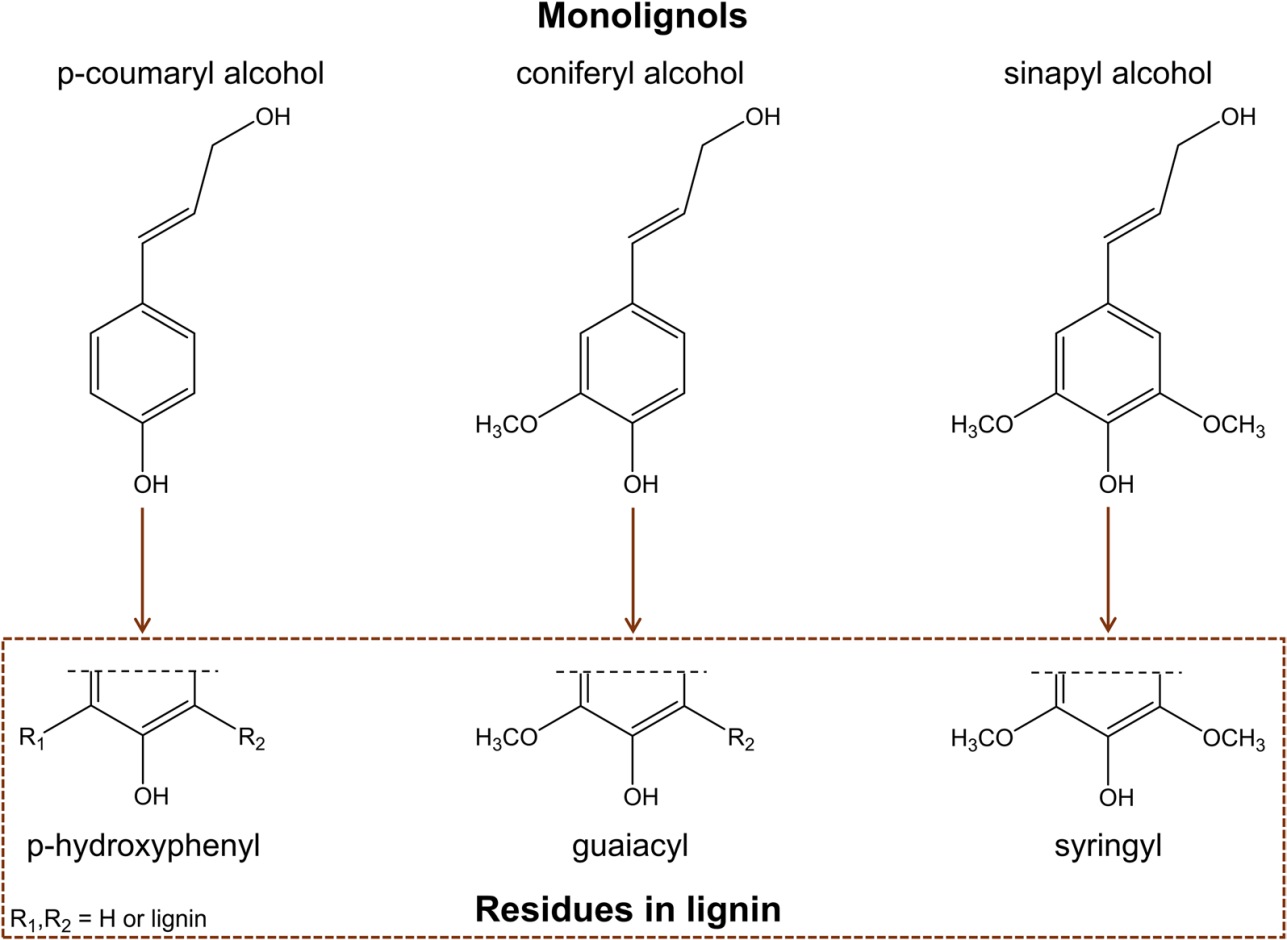
The three major precursor monomers and their resultant structure in lignin. Illustration adapted with permission from [13]. Copyright 2014, Elsevier

**Fig. 2:**
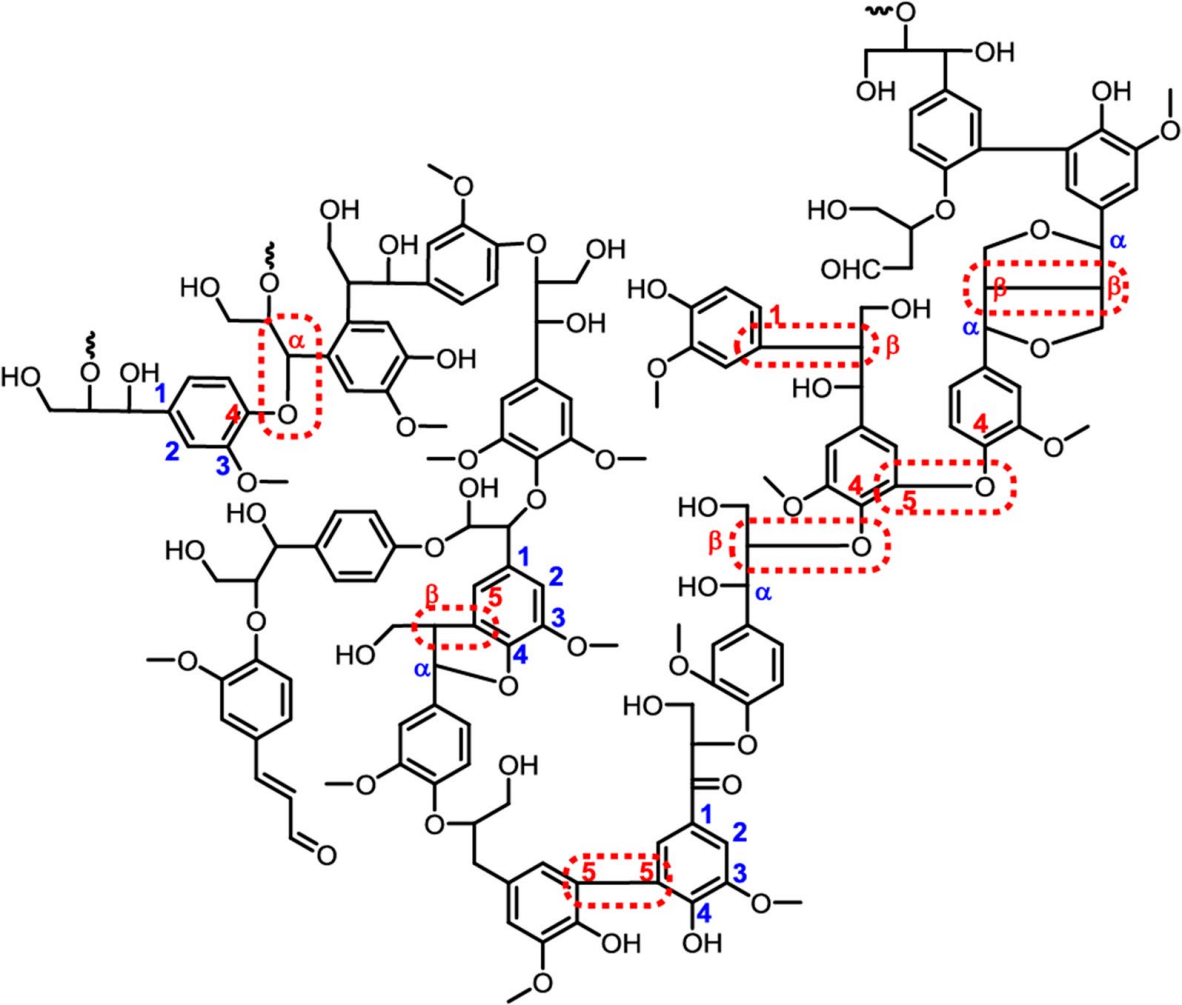
Schematic structure of lignin with common interunit linkages. Reprinted with permission from [4]. Copyright 2016, Elsevier

The chemical composition of lignin comprises a random network of phenylpropane units referred to as p-hydroxyphenyl (H), guaiacyl (G), and syringyl (S) [5, 6], respectively, as illustrated in Fig. 1. Lignin heterogeneity arises from the different concentrations of these monomers depending on the origin and growing conditions of the plant. Softwood species mainly contain G-units, whereas hardwoods have G- and S-units, with the latter being predominant. For herbaceous plants, the three units are present [7, 8]. The interactions between the units generate different interlinkages, with the major ones being carbon–carbon (C–C) and ether (C–O–C) linkages [9]. Typically, the most abundant lignin interunit is the β-aryl ether type (β-O-4), representing about 50% and 60% of total linkages in softwoods and hardwoods, respectively [1, 8]. Other relevant linkages and proximate proportions are α-O-4 (2 to 8%), β-5 (6 to 12%), 5–5 (5 to 11%), 4-O-5 (4 to 7%), β-1 (7%), β-β (2 to 3%) [4, 10, 11] (Fig. 2). Within the lignin phenylpropane units, several functional groups are present, including aromatic methoxyl and phenolic hydroxyl, primary and secondary aliphatic hydroxyl, small amounts of carbonyl groups, and carboxyl groups. The presence and quantity of these groups impact the lignin chemical reactivity, optical and dispersion characteristics, and functional properties [4, 12].

Furthermore, the lignin extraction from lignocellulosic biomass significantly influences its structure, altering the original linkages and functional groups [3]. The main conventional lignin extraction processes, namely the Kraft and sulfite pulping, originate from the pulp and paper industry [7]. The Kraft process subjects wood to sodium hydroxide and sodium sulfide at temperatures of 150–170 °C, yielding a cellulose-rich solid and a lignin-containing black liquor. During pulping, lignin suffers several cleavages in the ether linkages and is converted to small fragments that become soluble in the black liquor [7, 8]. Kraft lignin is recovered through acidification and precipitation of the black liquor [8, 14, 15]. The severe alkaline conditions result in a highly modified lignin with an increased phenolic hydroxyl content, high polydispersity, and sulfur levels ranging from 1 to 3% as thiols, which impart a characteristic odor [4, 13, 14].

In contrast, the sulfite process delignifies wood using sulfurous acid salts (e.g., calcium, sodium, magnesium, ammonium) at 125–150 °C and pH 1–5 [4, 7, 13]. This treatment cleaves lignin, yielding sulfonated derivatives (lignosulfonates) containing carbohydrates, wood extracts, and inorganic residues [14, 15]. Lignosulfonates are water-soluble due to their high sulfonate content, exhibit a broad molecular weight distribution, and typically have a high ash content [4]. Another well-established extraction process is the organosolv process, which utilizes mixtures of organic solvents and water to fractionate the biomass at several temperatures (80–250 ºC) and pressures [7, 14]. The process extracts high-purity lignin with a low molecular weight and retains most of the cellulose, making it a promising technique in emerging biorefineries [8, 16].

Approximately 100 million tons of technical lignin are generated annually, with the majority currently used for internal energy input [17, 18]. However, approximately 40% of the extracted lignin is enough to fulfill the intern industrial energetic demand [19]. As a result, lignin has gained attention as a valuable feedstock for producing renewable chemicals and materials, offering a sustainable alternative to petroleum-based resources [17, 20]. Table 1 summarizes key players in the lignin market. Currently, lignosulfonates are the predominant type due to their well-established industrial applications, including dispersants, resins, and binders [17, 20, 21]. Major producers include Borregaard LignoTech, Rayonier Advanced Materials Inc., and Domsjö Fabriker AB. Kraft lignin, the most abundant source of technical lignin, is starting to be commercialized for products other than bioenergy, with an expected market of 3.5–14 million tons/year [17, 20]. The increasing interest in lignin valorization by kraft pulping industries is related to improvement in the pulping process associated with more efficient and less costly kraft extraction technologies [22]. Companies such as Suzano and Stora Enso are contributing to this shift toward value-added lignin streams through innovative technologies and pilot-scale production facilities.

**Table 1:** Overview of lignin suppliers, production, and applications

Lignin supplier	Lignin source	Extraction type	Production capacity (Kt/year)	Applications	References
Bloom Biorenewables	Softwood Hardwood Agricultural side-streams	Aldehyde-assisted lignocellulose fractionation	0.05	Cosmetics, homecare, resins, adhesives, elastomers, bioplastics	[25]
Borregaard LignoTech	Softwood Hardwood	Sulfite	1000	Dispersants, binding agents, stabilizers	[26]
Domsjö Fabriker AB	Softwood Hardwood	Sulfite	120	Concrete, oil, carbon black, binders, agrochemicals	[27, 28]
Fibenol	Low-quality hardwood and plywood industry residues	Sunburst pre-processing fractionation	6.5	Coatings, plastics, composites, foams, cosmetics, resins	[23]
Ingevity	Softwood Hardwood	Kraft Sulfite	60	Dispersants, emulsifiers, fertilizers, binders, resins, carbon fibers	[28]
Rayonier Advanced Materials Inc	Softwood Hardwood	Sulfite	150	Fibers, biopolymers	[28]
Stora Enso	Softwood Hardwood	Kraft	50	Binder, batteries, composites, resins	[29]
Suzano	Hardwood	Kraft	20	Elastomers, resins, cosmetics, carbon materials, adhesives, animal feeding	[30]
UPM	Softwood Hardwood	Kraft	1	Resins, adhesives, plastics, polyurethanes, fillers	[28, 31]

In addition, increasing interest in lignin valorization for high-value applications (e.g., resins, carbon fibers, bio-based chemicals, cosmetics, and personal care) has driven innovation in lignin extraction and purification technologies. Fibenol, an Estonian biorefinery, employs its proprietary Sunburst™ pre-processing technology to fractionate forestry and wood industry residues into high-purity lignin and cellulosic wood sugars, which serve as sustainable feedstocks for the production of biochemicals and biomaterials [23]. Another example is Bloom Biorenewables, a Swiss company that utilizes an aldehyde-assisted fractionation (AAF) process to selectively stabilize and separate lignin from lignocellulosic biomass, resulting in uncondensed, highly pure lignins with a well-defined molecular structure and light brown coloration, suitable for high-value applications in materials science, specialty chemicals, and sustainable polymers [24, 25].

## Colloidal lignin particles (CLPs)

According to the International Union of Pure and Applied Chemistry (IUPAC), colloidal particles are particles dispersed in a medium, with at least one of their dimensions ranging approximately between 1 nm and 1 µm [32]. In contrast, nanoparticles are usually defined as particles in the size range between 1 and 100 nm, however, many authors use this term to refer to particles up to 1000 nm in size [33–37]. Since no consensus has yet been reached regarding the boundary between the two classes [2, 32], in this work the terms particles, colloidal particles, and nanoparticles are used as defined by the respective authors in their original studies.

### Production

The direct use of lignin for high-value applications is hindered by its heterogeneous structure and limited compatibility with aqueous systems [38]. A straightforward approach to address these challenges is the development of CLPs. In this form, lignin adopts a more organized structure and exhibits significantly improved dispersibility in water-based systems. Furthermore, their high surface-area-to-volume ratio and well-defined morphology enhance lignin's functional properties, such as antioxidant and antimicrobial activities, UV protection, and thermal stability [2, 20, 39]. Consequently, CLPs are considered a practical approach to unlocking lignin's full potential, surpassing the limitations of its native macromolecular form [1, 2].

One of the main advantages of CLPs production is that it does not involve lignin derivatization by chemical routes, thus requiring less energy and not generating chemical wastes [2]. Frangville and co-workers were the pioneers in producing lignin particles in 2012, following two methodologies: (1) precipitation from ethylene glycol solution by using diluted acidic aqueous solution followed by dialysis and (2) a pH change precipitation from basic to acidic aqueous medium [34]. Based on Frangville's work, several studies emerged with new approaches for producing CLPs. Currently, the most used methods are based on lignin precipitation by the presence of an antisolvent [11]. Typically, lignin is dissolved in a suitable organic solvent (e.g., tetrahydrofuran, ethylene glycol, or acetone), after which an antisolvent is added to reduce lignin's solubility and induce precipitation. The chosen antisolvent must be miscible with the organic solvent but immiscible with lignin (e.g., water) [1, 2]. This perturbation prompts lignin's rearrangement into particles to minimize the surface area in contact with the antisolvent [2]. This process can also be achieved through pH changes in an aqueous medium, as lignin can be dissolved in highly basic solutions (pH > 11) and precipitated by reducing the pH [34]. However, acidifying the lignin solution causes protonation, which leads to particle formation that inevitably sediments over time, resulting in poor storage stability [2]. The most common techniques to produce lignin particles are summarized in Table 2, including their advantages and disadvantages.

**Table 2:** The main production methods of lignin particles [1, 2, 19, 40, 41]

Method	Description	Advantages	Disadvantages
Acid precipitation	Lignin is dissolved in a high pH aqueous solution and precipitated by the addition of an acidic solution	– Simple methodology – Controllable size and morphology – High particle yield	– Poor storage stability
Antisolvent precipitation	Lignin is solubilized in an organic solvent, followed by the addition of an antisolvent (typically water)	– Controllable size and morphology – Low-cost antisolvent (water) – High particle yield	– High cost and toxicity of some organic solvents
Solvent shifting/exchange	Lignin is solubilized in an organic solvent, and the solution is placed into a dialysis bag, which is immersed in excess water	– Controllable size and morphology – Low-cost antisolvent (water) – High particle yield	– High cost of some solvents and dialysis membranes – High water consumption – Toxicity of some organic solvents – Difficulty in solvent recovery – Long preparation time
Ultrasonication	Sonication of lignin aqueous suspensions for a certain time	– Single-step process – Fast synthesis – Low cost	– Broad size distribution – Irregular shape and morphology
Aerosol flow	Lignin is dissolved in an organic solvent, followed by atomization into particles, which are suspended and transported by a carrier gas through a heated laminar flow system and collected downstream in the system	– Single-step process – High particle yield – Absence of liquid by-products – Continuous operation	– High energy consumption – Toxicity of some organic solvents – Poor storage stability

### Factors affecting the formation of CLPs

The formation of CLPs is primarily driven by the amphiphilic nature of lignin, where its hydrophobic segments self-assemble in the core and the hydrophilic portions become oriented toward the particle surface [1]. The main driving force for this behavior is its natural tendency to reach an equilibrium state after the addition of an antisolvent from a non-equilibrium state. Thus, the surface of the lignin particles is formed through hydrophilic aggregation and stabilized by non-covalent interactions such as hydrogen bonding involving the hydroxyl groups positioned at the surface. On the other hand, the core is formed through hydrophobic aggregation stabilized by noncovalent interactions, such as hydrophobic interactions in the presence of water [1, 2, 40]. In addition, two levels of aggregation are reported to occur in the process: molecular aggregation of polymer chains through van der Waals interaction and π-π aggregation between aromatic rings [1, 41, 42].

Depending on the applied methodology, several process variables are involved in the particles' formation, which impact their final characteristics, such as stability, size, and morphology [34]. In the antisolvent precipitation technique, it has been reported that the initial lignin concentration influences the aggregation and final particle size [34, 37, 43, 44], where a high initial lignin concentration results in increased particle sizes due to the high lignin availability to assemble into larger particles during formation [44]. After reaching a specific initial concentration threshold, the particles form aggregates, leading to sedimentation. For example, this limit was achieved at approximately 20 mg/mL in Lievonen's process [44], while Sipponen et al. [43] reported particle aggregation at an initial lignin concentration above 3 mg/mL.

**Fig. 3:**
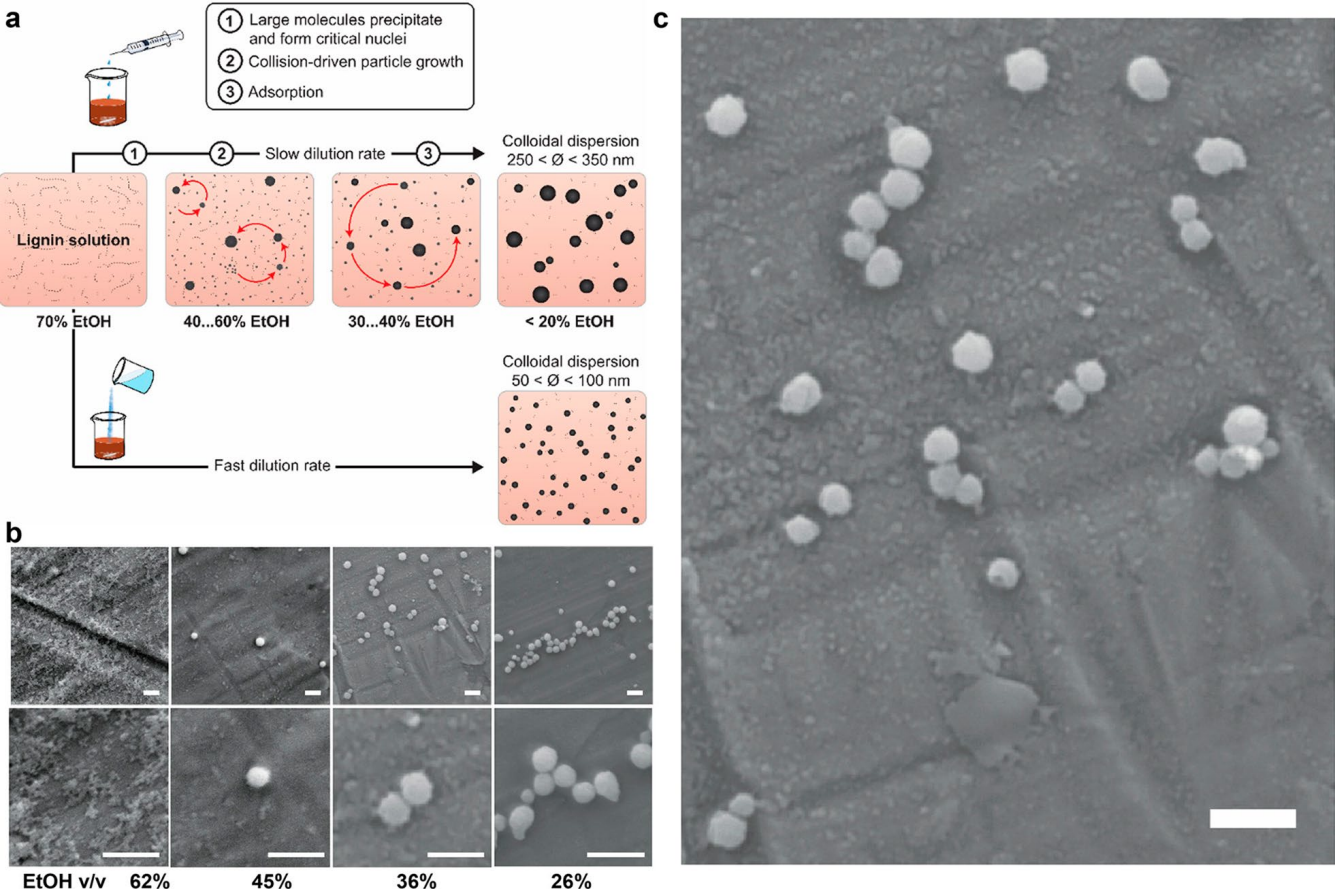
Schematic representation of colloidal lignin particle formation upon addition of antisolvent and decrease of the solvent concentration in the medium. Reprinted with permission from [43]. Copyright 2018, American Chemical Society

The antisolvent volume and addition rate are also key factors in forming CLPs. Typically, a faster antisolvent addition rate leads to smaller particles, as there is less time available for particle growth [45]. However, beyond a certain threshold, a further increase in the antisolvent addition rate does not reduce particle size, indicating that the maximum number of nuclei has been formed [35]. Similarly, the antisolvent volume, which can also be analyzed in terms of the final concentration of the organic solvent in the medium [43, 46], plays a crucial role in lignin particle formation. Upon addition of antisolvent in the medium, the lignin solubility is reduced, thereby directly affecting the final particle characteristics. Sipponen et al. demonstrated that lignin precipitation upon the addition of water (antisolvent) to a 70% ethanol lignin solution occurred from high to low MW fractions [43]. More specifically, large molecules precipitated at ethanol concentrations of 60–40%, forming critical nuclei with sizes around 60 nm. With an ethanol concentration decrease of 30–40%, the nuclei grew through the aggregation of intermediate particles. At an ethanol concentration of 13%, a few small particles precipitated, with sizes lower than 50 nm, but most particles were larger due to the growth of the initial nuclei to a size range of 250–350 nm [43]. The schematic representation of this mechanism is shown in Fig. 3.

The antisolvent pH is another variable that affects the formation and stabilization of the particles through electrostatic interactions. However, this parameter is often neglected, with few works addressing this variable [46–48]. Leskinen et al. used NaOH aqueous solutions as antisolvent at different base concentrations (0.1 to 6 mM). They observed a size reduction of the particles with increased base concentration, owing to the improvement of the electrical double-layer repulsion [46]. This contributed to a decrease in lignin agglomeration during particle formation, suggesting that increasing the pH of the antisolvent enables the production of CLPs at higher lignin concentrations [46], which may be an advantage for the design of a productive process.

Intrinsic lignin properties, such as lignin type and molecular weight, also influence the final characteristics of CLPs [37, 49]. However, despite various efforts to correlate the structural properties of lignin with the final morphology and size of CLPs, no clear consensus has been reached in the literature [37, 50–52]. Some studies have reported a dependence on molecular weight, where CLP size decreases as the lignin MW increases, likely due to the faster nucleation associated with the high MW fractions [50–52]. In contrast, Lee et al. [37] observed the opposite trend for CLPs derived from soda lignin fractions obtained via sequential organic solvent extraction. Additional structural parameters were investigated by Sapouna et al. [50] in CLPs prepared from native-like hardwood and softwood lignin fractions. They found that samples with a high β-O-4’ content formed smaller particles, possibly due to the higher flexibility of these linkages, which allows for a higher degree of packing. Furthermore, they concluded that the monolignol content had a stronger influence on particle morphology than either β-Ο-4’ content or lignin concentration. Despite these findings, no definitive structure–property relationship has been established.

Contributions to this topic have also been made by Pylypchuk et al. [52–54]. They found that an increasing number of aliphatic hydroxyl groups correlated with a decrease in lignin particle size when using eucalyptus and spruce lignin. This was likely due to the ability of aliphatic hydroxyl groups to form strong intramolecular hydrogen bonds, which promote molecular folding rather than intermolecular aggregation. As a result, lignin–lignin interactions are reduced, clustering is limited, and smaller particles are formed [54]. Furthermore, they found that softwood lignin formed smaller particles than hardwood lignin and attributed this behavior to stronger π–π interactions in G units compared to S units. These stronger interactions promote denser molecular packing, leading to smaller sizes in softwood-derived lignin particles, where G units are predominant [54]. In contrast, Colucci et al. [51] found the opposite trend, where CLPs from the hardwood-type samples presented smaller sizes than those from the softwood-type samples. The authors elucidated that the higher total OH content of softwood-type samples, compared to hardwood-type samples, suggests that the former are more hydrophilic, thus yielding larger particles due to higher affinity with aqueous medium and, consequently, a less dense packed structure. Additionally, differences in the initial lignin concentration and in the techniques employed in the various studies may have also contributed to the contrasting results, as both factors can influence particle size patterns.

Overall, the most evident conclusion is that the MW determines the size of lignin particles for a given lignin type (i.e., lignin from a particular source and extraction process), which is attributed to the overall nucleation kinetics during particle formation [52]. However, when comparing lignins of distinct types and proceeding from different extraction processes, additional structural factors influence particle size. Indeed, the numerous variables in lignin's structure make it challenging to identify the key parameters influencing CLP formation. This challenge may be addressed by developing standardized methods and novel experimental protocols that are better suited for characterizing the complex structural properties of lignin.

## Pickering emulsions

Emulsions stabilized by solid particles are known as Pickering emulsions, named after S.U. Pickering, whose pioneering work in 1907 described the formation of oil-in-water (O/W) emulsions stabilized solely by solid particles [55]. Although discovered over a century ago, Pickering emulsions have attracted significant interest only recently, following advances in materials science and the development of new materials suitable as Pickering stabilizers [56, 57]. The major advantage of Pickering emulsions when compared to conventional emulsions is their high stability against coalescence, owing to the irreversible adsorption of particles at the oil–water interface [56–58]. Furthermore, they also tend to be less toxic, more cost-effective, and easier to recover than synthetic emulsifiers [59–61]. Over the past decade, there has been an exponential increase in publications and patents related to Pickering emulsions, reflecting a growing interest in their potential for commercial applications [62, 63]. For example, Pickering emulsions have been designed for use in various applications, including food [64–66], cosmetics [67–70], pharmaceuticals [71–73], catalytic processes [74, 75], and filtration membranes [76, 77], among others [58, 78, 79].

### Parameters governing Pickering emulsion formation

The stabilization mechanism of Pickering emulsions is governed by the free energy of desorption (E_D_), which is the energy required to remove a spherical solid particle from the oil–water interface, as described in Eq. 1 [58, 59]:

1 $${E}_{D}(J)=\pi {r}^{2}{\gamma }_{ow}{\left(1\pm \mathrm{cos}{\theta }_{ow}\right)}^{2}$$

where *E*_*D*_ is energy of desorption given in Joule, *r* is the particle radius, *γ*_*ow*_ is the oil–water interfacial tension, and *θ*_*ow*_ is the three-phase contact angle between the solid particle, the oil, and the water phases [58, 78]. From Eq. 1, it can be noted that the wettability, the particle size, and the oil type are important factors governing the formation and stabilization of Pickering emulsions [63].

**Fig. 4:**
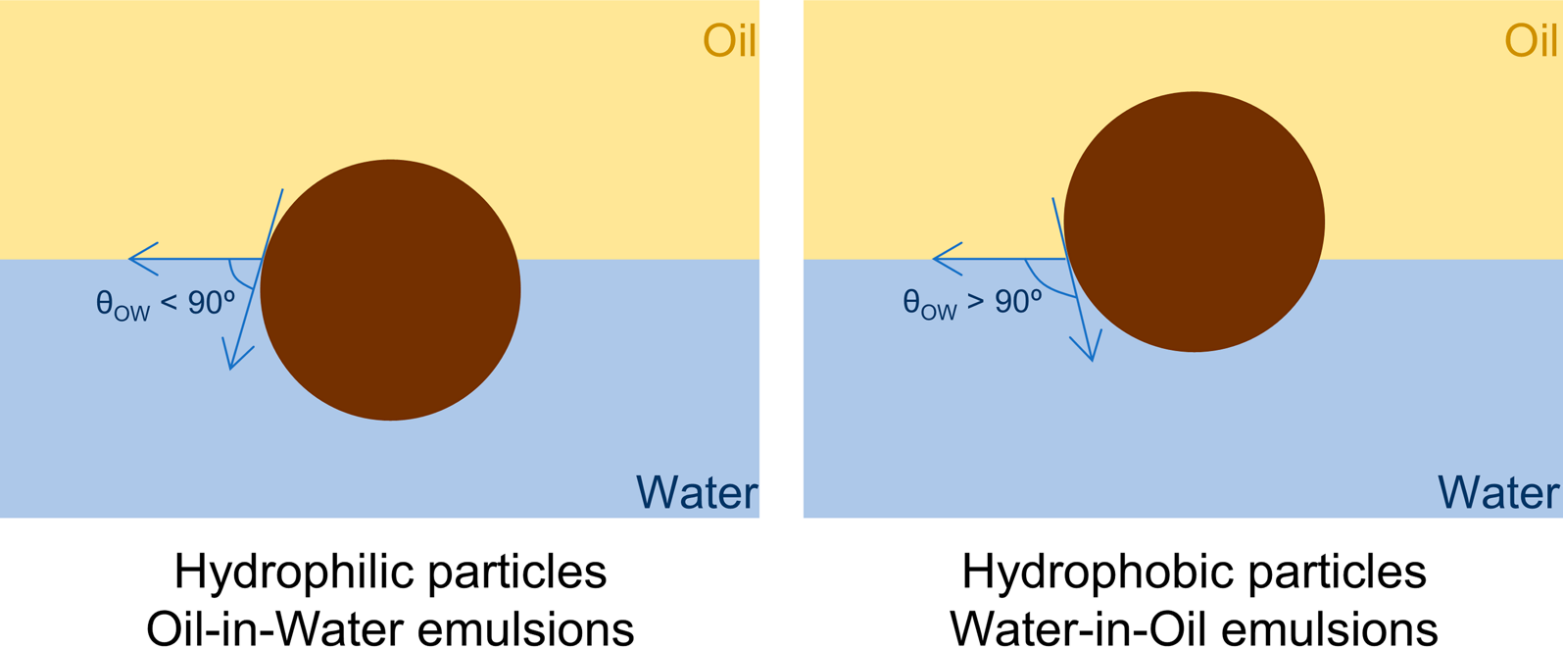
Particle’s wettability represented by the three-phase contact angle *θ*_*OW*_. Illustration adapted with permission from [58]. Copyright 2019, Elsevier

Regarding particle wettability, an important requisite for particles to act as Pickering stabilizers, they must be partially wetted by both water and oil phases [57]. Conventionally, particles with 15º < *θ*_*ow*_ < 90º are hydrophilic and preferentially stabilize O/W emulsions whereas particles with 90º < *θ*_*ow*_ < 165º are hydrophobic and preferentially stabilize W/O emulsions (Fig. 4). When the *θ*_*ow*_ is equal or close to 90°, particle adsorption at the interface is maximized, indicating its strong anchoring at the oil–water interface, and thus, highly stable emulsions are formed [58, 80, 81]. At *θ*_*ow*_ values outside these ranges, the particles are either too hydrophilic (close to 0º) or too hydrophobic (close to 180º) to be adsorbed at the oil–water interface [81]. Nonetheless, some authors claim different ranges for closely packed particles or particles with 3D networks. For these cases, particles with 15º < *θ*_*ow*_ < 129.3º can effectively stabilize O/W emulsions whereas particles with 50.7º < *θ*_*ow*_ < 165º are appropriate to stabilize W/O emulsions [81, 82]. The emulsion type formed at the overlapping interval (50.7º-129.3º) depends on the oil volume fraction (ɸ) and preparation conditions [58, 81].

The particle size has a relevant impact on emulsion stability and droplet size. From Eq. 1, the larger the particle size, the higher is the energy required to remove it from the interface, indicating that stable emulsions are obtained with larger particles. However, Li et al. showed that it was impossible to produce emulsions when large potato starch particles were used [83]. As the desorption energy is higher for larger particles, the adsorption energy is also higher, and from this point of view, small particles would produce more stable emulsions due to the faster adsorption kinetics [63]. Binks and Lumdson showed that emulsions produced with small particles were more stable against sedimentation [84]. Thus, it is clear that particle size plays an important role in the emulsion stability, although there are, possibly, other forces affecting this property [63]. Regarding the emulsion droplet size, a linear correlation is observed, whereby the droplet size decreases with the particle size. In general, the solid particles must be one order of magnitude smaller than the desired droplet size, in order to produce stable emulsions [57, 63].

**Fig. 5:**
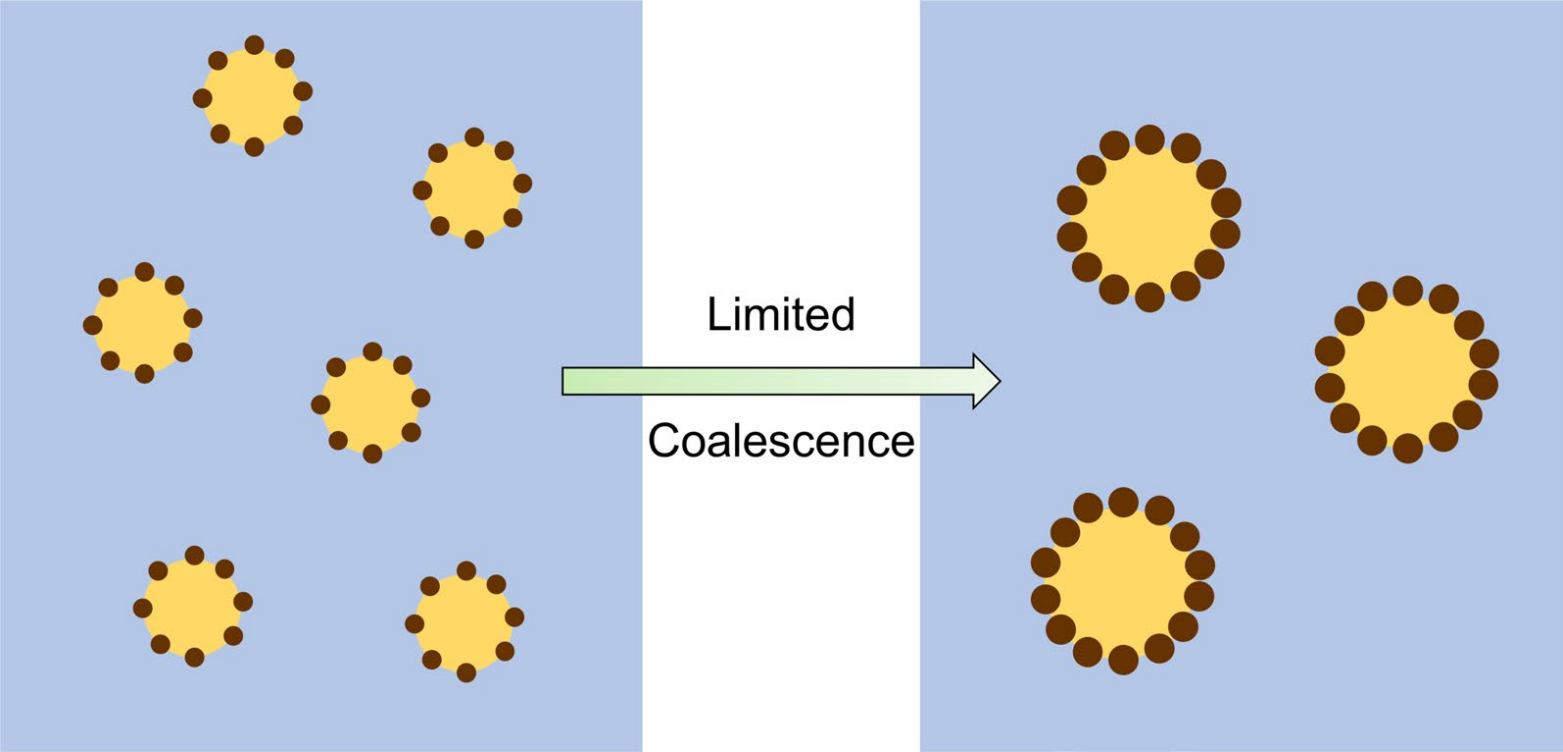
Representation of the limited coalescence phenomena in Pickering emulsions. Illustration adapted with permission from [58]. Copyright 2019, Elsevier

Another relevant parameter in Pickering emulsions formation is the particle concentration, in which three distinct regimes are observed [57]. When a low concentration of particles is used, the droplets coalesce before being stabilized by the particles. At an intermediate concentration, where the interfacial area produced during emulsification is slightly larger than that the particles can stabilize, the droplets will undergo a “limited coalescence” (Fig. 5) until reaching a droplet diameter where they become fully covered by particles [57, 58, 85]. At high particle concentrations, fine emulsions can be produced if sufficient emulsification energy is applied to generate the total interfacial area that particles can cover. Otherwise, only part of the particles is adsorbed, and the excess remains in the dispersed phase [58]. At this point, emulsion thickening may occur due to the self-aggregation of solid particles in the water continuous phase, if the particles are not too hydrophilic to be dispersed. This is a specific feature of Pickering emulsions, which typically present a gel-like structure, providing an advantage from a stability perspective. The high viscosity decelerates instability phenomena, and in extreme conditions where emulsions are gelled, long-term stability is observed [57, 86, 87].

The oil chosen to be dispersed is directly related to the interfacial tension between water and oil, influencing the E_D_ and* θ*_*ow*_, which in turn is related to the stability and emulsion type [78]. For example, Tan et al. studied the formation of emulsions stabilized by gelatin nanoparticles with either corn oil or medium-chain triglycerides (MCT) [88]. They found that MCT oil emulsions were more stable owing to the formation of denser nanoparticle layers at the interface. In contrast, the surface-active components of corn oil competed with gelatin nanoparticles for accumulation at the interface, resulting in a sparse packing interface and less stable emulsions [88].

Although not considered in the latter equations, the oil-to-water ratio is a significant parameter in emulsion stability, as it dictates the amount of dispersed phase and thus the available interfacial area to be covered by the particles [63]. Sharkawy et al. produced olive oil Pickering emulsions with chitosan/Arabic gum nanoparticles and observed an increased stability starting at oil/water ratios greater than 0.5, whereas creaming was detected for oil/water ratios lower than 0.5, for a fixed nanoparticle concentration of 1.5% (w/v) [89]. It was suggested that the oil volume fraction increase lead to an increased emulsion viscosity, which could contribute to the higher stability of the emulsion system [89].

### Stabilization mechanisms

**Fig. 6:**
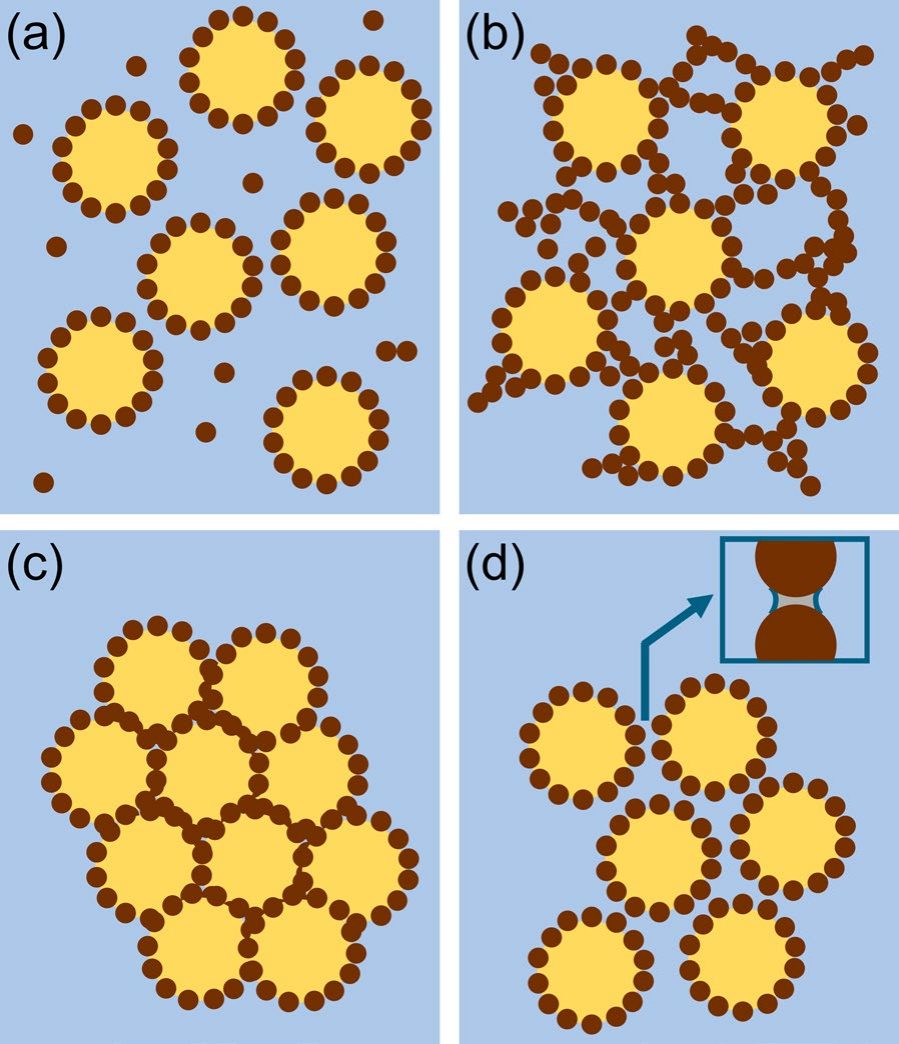
Stabilization mechanisms of Pickering emulsions: **a** interfacial membrane barrier, **b** three-dimensional grid, **c** particle bridging, and **d** capillary force. Illustration adapted with permission from [59]. Copyright 2023, Elsevier

The formation of Pickering emulsions during emulsification typically involves four key steps: (i) droplet formation induced by shear forces, (ii) approach and collision of the particles with the droplets, (iii) adsorption of the particles at the oil–water interface, and (iv) formation of a particle network within the continuous phase [59]. Depending on the interactions between particles and the oil/water phases, as well as between the particles themselves, various stabilization mechanisms may occur either independently or simultaneously (Fig. 6).

The most typical stabilization mechanism of Pickering emulsions is known as the interfacial film barrier mechanism, where particles adsorb at the oil–water interface, forming an interfacial film that prevents droplet contact and interaction at the interface. In this mechanism, particles are arranged in a single layer or multilayer that sterically hinders the coalescence of the emulsion droplets [59, 90].

Lagaly et al. introduced the concept of a three-dimensional network mechanism in their studies on clay particles [91]. This theory suggests that solid particles dispersed in the continuous phase can organize into a 3D network surrounding individual droplets. This structure limits the mobility of the droplets, effectively preventing phase separation between oil and water. Additionally, the network contributes to increased system viscosity, thereby reducing droplet coalescence and aggregation, which enhances the stability of Pickering emulsions [59, 92]. For this mechanism to function effectively, a sufficiently high particle concentration and strong interparticle attraction within the continuous phase are essential [59].

Another observed mechanism in Pickering emulsions is the bridging mechanism, where the droplets are stabilized by a shared particle monolayer network that exerts strong adhesive forces, preventing droplets from aggregating [93]. The essential prerequisites for particle bridging include a high affinity of the particles with the continuous phase as well as a large interfacial area generated during the emulsification process [94]. The particle bridge exhibits strong affinity for both connected droplets, effectively keeping them apart and inhibiting their aggregation or coalescence [59].

The mechanism of capillary forces arises due to the formation of a liquid meniscus between two solid surfaces, creating an attractive interaction [59]. At the interface, these forces tightly arrange adsorbed particles in sequence, thereby minimizing the risk of droplet coalescence [59, 92]. For particles ranging from the nanoscale to the microscale, wettability plays a key role in enabling capillary interactions. Furthermore, anisotropic particles (e.g., rods, disks, or fibers) induce distortions at the interface and are more prone to generate capillary attraction forces than spherical particles [59, 92].

### CLPs in Pickering emulsion stabilization

Owing to their partial wettability at oil–water interfaces, CLPs can assemble into associative structures that stabilize emulsions by adsorbing at the interface, providing electrostatic and steric repulsion, and forming an interfacial film around droplets that inhibits coalescence. In this regard, CLPs are emerging as innovative Pickering stabilizers [95–97].

Scopus database shows an increase in publications of Pickering emulsions stabilized by lignin particles, in which a total of 118 articles were found for the search TITLE ("lignin") AND (TITLE-ABS-KEY ("Pickering") OR TITLE ("Pickering")) with the last years (2024 and 2025) having the highest numbers of publications. In this search, the work of Wei et al. [98] in 2012 is the first to address Pickering emulsions stabilized by CLPs, in which alkali lignin was extracted from furfural production residues proceeding from the acid degradation of plants such as corncobs and wheat straw. CLPs were prepared by acid precipitation and subsequently used to stabilize styrene emulsions, which remained stable at pH values lower than 4 but exhibited completely separated phases at pH values higher than 10. This work demonstrated the ability of lignin particles to stabilize pH-responsive emulsions, which can be applied to various areas, including emulsion polymerization [98]. Other studies have emerged since then, corroborating the potential of lignin to act as a Pickering stabilizer. Table 3 summarizes some of the studies published on the topic, presenting information about the lignin particle (lignin type/modification), the emulsion components (oil phase and particle concentration), preparation method, and intended application.

**Table 3:** Literature review on Pickering emulsions stabilized by lignin particles

Particle composition	Particle concentration^a^ (mg/mL)	Oil phase	Oil volume fraction	Production technique	Final application	Refs.
Softwood Kraft lignin	3.5–35	Orange, coconut, and paraffin	0.1–0.5	Ultraturrax (10,000 rpm, 2 min) and Sonication	Skincare	[99]
Grass soda lignin	25	Sweet almond oil	0.1	Ultraturrax (10,000 rpm, 10 min)	Not specified	[100]
Alkali lignin (alkylated)	2.5	Canola oil and cinnamaldehyde	0.3	Sonication	Biomedical and antibacterial	[101]
Lignin-containing cellulose	10	Canola oil	0.1	Ultraturrax (30,000 rpm, 3 min)	Food preservation	[102]
Lignin from cocoa shell	2	Sunflower oil	0.1	Ultraturrax (9000 rpm, 2 min)	Food	[103]
Enzymatic hydrolysis lignin and chitosan composites	100–180	Soybean oil and curcumin	0.8–0.85	Ultraturrax (16,000 rpm, 1 min)	Combination chemotherapy	[104]
Alkali lignin	0.5, 1, 2, 4	Limonene, styrene, methyl methacrylate, n-tetradecanol, n-hexadecane, or dichloromethane	0.1–0.9	Ultraturrax (9,000 rpm, 3 min)	Not specified	[105]
Kraft lignin coated with chitosan and glucose oxidase	0.25–5	Styrene	0.2	Sonication (2 min)	Template to form nanocomposites	[106]
Alkali lignin	2	Cinnamaldehyde, eugenol, or allyl isothiocyanate	0.1	Sonication (1 min)	Antimicrobials for food preservation	[107]
Sulfoethylated Kraft lignin	2.5	Xylene	0.5	Sonication (30 s)	pH-responsive emulsions for industrial applications	[108]
Amphoteric alkali lignin	0.1–10	Soybean oil, n-decane, or paraffin	0.5	Ultraturrax (11,000 rpm, 3 min)	Drug controlled release, oil–water separation, and functional materials	[109]
Kraft lignin	2, 4, 8	IPDI and n-eicosane	0.1–0.3	Ultraturrax (9,000 rpm, 3 min)	Template for microcapsule synthesis	[110]
Lignin from BLN process (Bioforce)	1.5–6	Canola oil	0.1	High-pressure homogenizer	Green chemistry	[111]
Kraft (Indulin AT) and organosolv	1–6	Kerosene	0.5	Sonication (1 min)	Not specified	[112]
Soda and organosolv	0.5	Olive oil, toluene or silicone oil, and curcumin	0.2	Vortex (1 min)	Encapsulating agents of bioactive compounds	[113]
Polymer-grafted soda lignin	1	Palm oil and trans-resveratrol	~ 0.35	Ultraturrax (10,000 rpm, 10 min) and sonication (30 s)	Food and biomedical	[114]
Polymer-grafted soda lignin	1	Palm oil and trans-resveratrol	~ 0.35	Ultraturrax (10,000 rpm, 10 min) and sonication (30 s)	Food and biomedical	[114]
Chitosan-coated Kraft lignin	2, 5, 10	Olive oil and ciprofloxacin	0.5	Sonication (1 min)	Controlled drug delivery	[115]
Kraft lignin	1, 2.5, 5, 10	Rapeseed oil	0.5	Vortex (30 s)	Not specified	[116]
Cationic Kraft lignin	1.25, 2.5, 3.75	Toluene, olive oil, or silicone oil	0.5	Vortex (1 min)	Lipase-catalyzed reactions	[117]
Kraft and organosolv	1–6	Kerosene	0.5	Sonication (1 min)	Not specified	[112]
Polymer-grafted Kraft lignin	1	Cyclohexane	0.1–0.9	Sonication (10 min)	Tunable Pickering emulsions	[118]
Enzymatic hydrolysis lignin	5, 10, 20, 40	Toluene	0.1 to 0.7	Shaking by hand	Template for the synthesis of molecularly imprinted polymers	[119]
Kraft lignin (Furfural residues)	0.5, 1, 5, 10	Styrene solution	0.2	Ultraturrax (12,000 rpm, 20 s)	Template for microcapsule synthesis	[98]

^a^ Particle concentration in the aqueous phase

Considering the great variety of lignins, several factors influence their ability to stabilize Pickering emulsions, including the molecular weight and type of functional groups, which are directly related to the lignin source and extraction method. This is also associated with lignin surface characteristics, namely wettability and electrostatic charge [95]. For example, Ago et al. [112] prepared lignin particles using an aerosol flow reactor with Kraft and organosolv lignins and utilized them to stabilize O/W emulsions. They reported that emulsions stabilized by organosolv lignins were less stable than those stabilized by Kraft lignins due to their lower hydrophilicity. This was accessed by the wettability of particles, in which Kraft lignin showed a contact angle with water of 57º whereas for organosolv the angle was 69º. They concluded that this feature contributed to interfacial rupture in the case of organosolv lignin-based emulsions, resulting in the release of oil into the water phase [112].

**Fig. 7:**
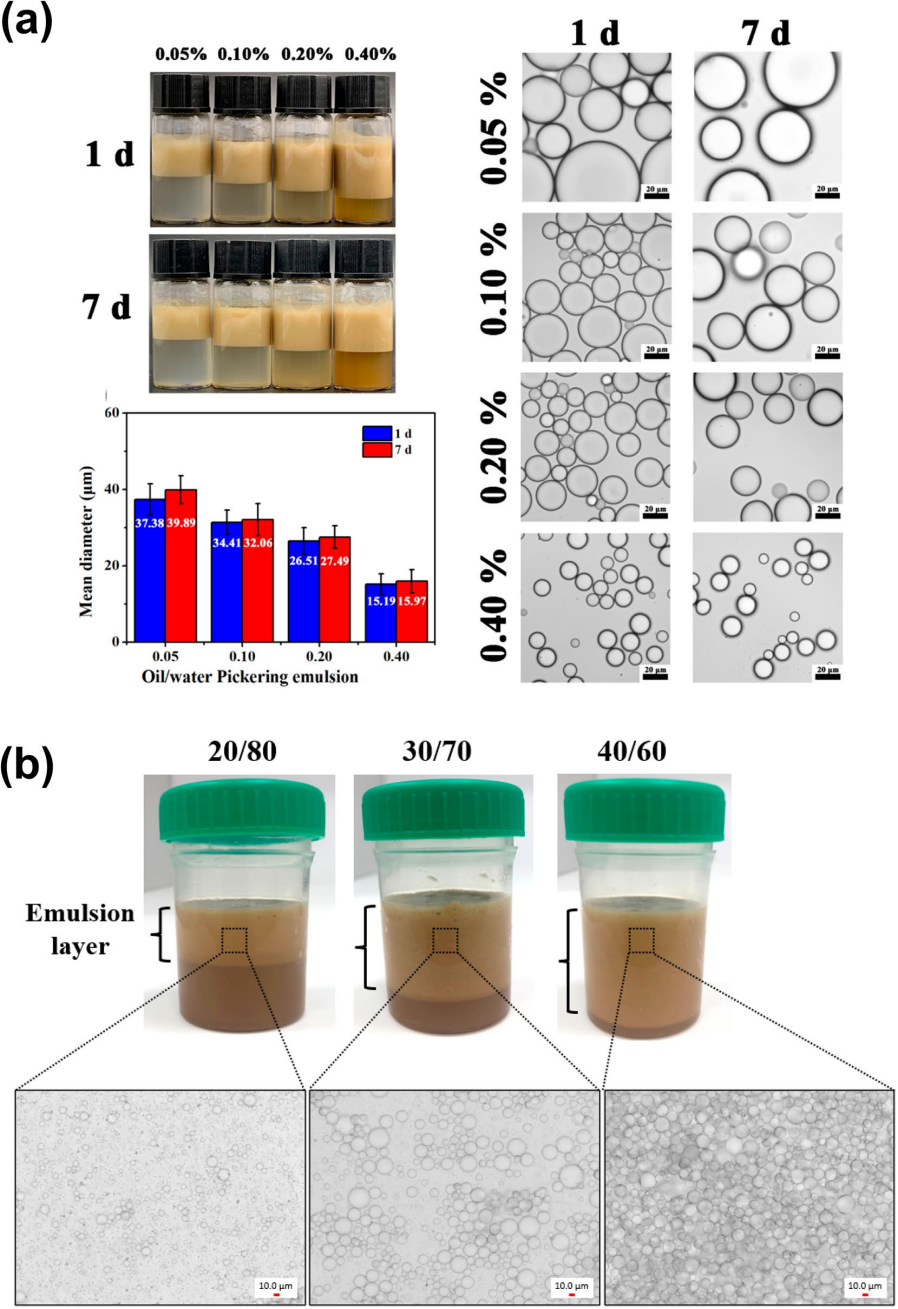
CLPs-stabilized Pickering emulsions: **a** with various lignin concentrations of 0.05, 0.10, 0.20, and 0.40 wt % prepared with thyme oil and **b** with various oil to water ratios (20/80, 30/70, and 40/60) prepared with parrafin oil. Reprinted with permission from [105] and [99], respectively. Copyright 2021, American Chemical Society and Copyright 2023, Elsevier

Li et al. [105] prepared thyme oil emulsions with lignin nanoparticles and observed that the oil droplet size decreased with an increase in particle concentration from 0.5 to 4 mg/mL, for a fixed oil volume fraction of 0.3 (Fig. 7a). This trend was also noted by Gan et al. [119], who prepared toluene emulsions varying lignin particle concentration from 5 to 40 mg/mL. This effect was justified by a larger interfacial area than the particles can cover at higher concentrations, leading to the formation of small oil droplets and enhanced stability due to the reduction of free energy. On the other hand, both studies showed that, for a fixed particle concentration in the aqueous phase, the oil droplet size increases as the oil volume fraction increases, as fewer particles are available to adsorb at the oil–water interface [105, 119].

Lignin-based Pickering emulsions have diverse applications, ranging from templates for the synthesis of materials to delivery systems for bioactive compounds. The use of lignin as a Pickering stabilizer for healthcare and pharmaceutical applications is still in its early stages; however, some studies have already shown positive results, motivating further research to explore this potential. An innovative approach was proposed by Chen et al*.* [104], which involved preparing a combined system containing both hydrophilic and hydrophobic anticancer drugs with lignin-based nanoparticles. For this purpose, they produced lignin/chitosan nanoparticles that encapsulated the hydrophilic drug and utilized them to stabilize O/W emulsions, with the oil phase containing the hydrophobic drug. In this sense, lignin nanoparticles not only acted as Pickering stabilizers but also as encapsulating agents. The developed delivery system presented a synergistic effect, in which the combined drugs exhibited improved performance against leukemia cells that were protected from UV, thermal, and oxidation damages [104]. This study demonstrates the potential of utilizing lignin in a diverse range of delivery systems.

For skincare applications, Gordobil et al. [99] prepared CLPs-stabilized Pickering emulsions using softwood kraft lignin and different oils (paraffin, orange, and coconut oils) to compare the UV protective effect of the formulations. They optimized emulsion formulation in terms of oil to water ratio (20/80, 30/70, and 40/60) and found that increasing the oil content increased the emulsion layer formed and contributed to higher stability, although the size of the droplets increased (Fig. 7b). They found that the Pickering emulsion system containing orange oil showed the best UV shielding performance, with a Sun Protection Factor (SPF) of around 13, owing to intrinsic properties of the oil.

Despite the promising results reported for CLP-stabilized Pickering emulsions, their long-term stability under the complex conditions characteristic of cosmetic formulations, such as variable pH and temperature, and potential ingredient interactions, remains largely unexplored and represents a significant knowledge gap in the field.

## Lignin for cosmetic applications

The growing demand for natural and organic cosmetics among consumers in recent years has significantly influenced the industry, driving the search for alternative ingredients to replace conventional synthetic compounds. Moreover, global environmental concerns are driving the development of strategies aimed at advancing a circular bioeconomy [120, 121]. In this context, lignin emerges as a potential carbon source, yielding relevant derivatives for the cosmetic industry, including vanillin and phenolic compounds. Besides, the direct use of lignin is gathering significant interest due to its remarkable intrinsic properties, such as antioxidant, UV-blocking booster, antimicrobial, biocompatibility, and biodegradability, making it a multifunctional ingredient for cosmetic formulations [122–125].

According to a search (September 2025) in the Scopus database using the terms “lignin” and “cosmetics” in article titles, abstracts, and keywords, the potential use of lignin in cosmetics yields approximately 535 publications. Research interest increased from 2011, with 2022–2024 as the years with the highest number of publications. Moreover, technological trends on lignin-based cosmetics (EspaceNet-database; words: lignin and cosmetic/cosmetics) indicated the first-published patent in 1987 (a cosmetic containing a lignin-degradation product) [126], and a recent invention (2020) granted to a sunscreen comprising lignin as the functional additive [127]. Additionally, it is worth highlighting LignoPure as a pioneering spinoff in the establishment of cosmetic-grade lignins, specifically LignoPure Base [128]. This evidence corroborates the growing technological and scientific interest in lignin-based cosmetics, encouraging the scientific community to pursue new developments.

As described in the previous section, lignin in the form of CLPs can function as a stabilizer in emulsion systems, thereby replacing key ingredients in cosmetic formulations such as creams, serums, and lotions. Due to its multifunctional nature, lignin may offer additional benefits within these formulations. The following subsections highlight the key properties of lignin that further support its application in cosmetic products.

### Antioxidant activity

Antioxidants are compounds that prevent oxidation reactions by inhibiting reactive oxygen species (ROS) [129]. ROS, such as hydroxyl (HO^•^), superoxide (O2^•−^), peroxyl (ROO^•^), hydrogen peroxide, and oxygen singlet, are responsible for causing oxidative skin damage, including aging, inflammatory responses, and DNA mutations [130–132]. These species are produced either intrinsically or in response to external factors, such as air pollution and UV radiation, the latter being reported as the primary route of ROS production. In this context, antioxidants are primarily included in cosmetic formulations to function as anti-aging, anti-inflammatory, and anti-carcinogenic agents [133]. Synthetic antioxidants, specifically butylated hydroxytoluene (BHT) and butylated hydroxyanisole (BHA), which are widely used, have been reported to have adverse effects on cells, even at low concentrations [129, 131, 134]. Therefore, there is an increasing demand for natural antioxidants that may replace the current ones applied in the cosmetic industry [133]. However, naturally sourced antioxidants are typically unstable molecules that degrade easily when exposed to light, oxygen, and heat, with vitamin C being a typical example [135].

Lignin has been considered a natural antioxidant due to its abundance in phenolic structures, which can neutralize reactive oxygen species (ROS) through electron donation and/or resonance effects. Its scavenging ability is dependent on the substituents of the aromatic ring and side-chain structures, in which hydroxyl and methoxyl groups play a crucial role in hydrogen donation and single-electron transfer reactions [130, 131, 136]. Studies have shown that lignin may present similar or higher antioxidant activity than commercial antioxidants [38, 137–139]. For example, Trevisan and Rezende [137] studied the antioxidant activity of lignin from elephant grass (in both crude and nanoparticle forms) using DPPH analysis and reported that both forms presented IC_50_ values lower than those of BHT. Furthermore, the lignin nanoparticles showed the highest inhibitory effect, with an IC_50_ of 12 µg/mL against 38 µg/mL for BHT, showing its potential to be an alternative antioxidant [137].

The stability of lignin and its particles under standard conditions provides an advantage over sensitive natural antioxidants. However, the structural heterogeneity of lignin remains a challenge for its commercialization as an antioxidant [140]. The source and extraction are important factors influencing lignin’s antioxidant activity as they directly impact its final structure and functional groups. Gordobil et al.[134] studied two types of Kraft and organosolv lignins, from spruce and eucalyptus sources. They firstly hypothesized the total phenolic content as a relevant factor influencing the antioxidant activity of lignins, as also stated in other studies [141–143]. However, the samples presented similar phenolic contents but different antioxidant activities, indicating that other molecular groups affected this property. The organosolv lignin from eucalyptus showed the strongest antioxidant activity, which was related to its high methoxyl content and low carboxylic acid content. Moreover, impurities such as ashes and carbohydrates were considered to have a negative effect on the antioxidant activity, whereas low molecular weight and narrow polydispersity contributed to increasing the activity, as also suggested by other studies [129, 134, 142].

The antioxidant activity of lignin and CLPs is usually measured using quantitative techniques based on radical scavenging or reducing power. Moreover, total phenolic contents, which correlate with antioxidant activity, can be determined for rapid screening of this activity using the Folin-Ciocalteu technique [144]. An overview of the most used methods is summarized in Table 4. They involve single-electron transfer (SET) and/or hydrogen-atom transfer (HAT) reactions, as described in Eqs. 2 and 3, respectively, where X is the radical, and AH is the antioxidant compound [129, 136].

**Table 4:** Common techniques applied to determine the antioxidant activity of lignin [136, 145–147]

Antioxidant assay	Mechanism	Description
DPPH (2,2-diphenyl-1-picrylhydrazyl)	HAT^a^/SET^b^	The compound’s ability to neutralize the DPPH radical by electron donation is accompanied by a color change of the DPPH radical, from deep purple to pale yellow. This color variation is measured by spectrophotometry at 517 nm
ABTS (2,2’-azino-bis (3-ethylbenzothiazoline-6- sulphonic acid)	HAT/SET	The antioxidant activity of a compound is determined by its ability to scavenge the stable radical ABTS, which has a blue-green coloration that decreases in intensity in the presence of antioxidants. The decrease can be measured by a spectrophotometer at 734 nm
FRAP (Ferric Reducing Antioxidant Power)	SET	This test is based on the antioxidant’s ability to reduce, under acid medium, ferric ions (Fe^3+^)-ligand complex to ferrous (Fe^2+^) complex, this later having an intense blue coloration. Antioxidant activity is determined as an increase in absorbance at 593 nm, and the results are expressed as micromolar equivalents of Fe^2+^ or to a standard antioxidant
FC/TPC (Folin-Ciocalteu-Assay or Total-Phenolics-Assay)	HAT/SET	This test is based on reducing the Folin–Ciocalteu reagent with phenolic compounds in an alkaline state. The reduction yields a blue-colored chromophore with maximum absorption at 765 nm, which can be measured by spectrophotometry. Gallic acid is the commonly used reference standard; thus, the results are commonly presented as Gallic acid equivalent weight (mg GAE/g)

^a^ HAT: hydrogen atom transfer

^b^ SET: single electron transfer

2 $$SET: X\bullet + AH \to {X}^{-}+A{H}^{+}$$

3 $$HAT: X\bullet + AH \to XH+A$$

To have more accurate data regarding the antioxidant activity, it is crucial to perform at least two different techniques, as they can present very distinct results. For example, Gordobil et al*.* [134] assessed the antioxidant activity of Kraft lignin and obtained significantly different IC_50_ values using the ABTS and DPPH techniques (3.47 and 16.63 µg/mL, respectively). The discrepancy was justified by the reaction rates of the tests, in which the ABTS radical reaction occurred at a much faster rate than the DPPH reaction. Table 5 summarizes studies from the literature that report the antioxidant activity of lignin in its crude or particle form, allowing for comparison of results obtained using different methodologies. Overall, the results suggest that lignin is a promising antioxidant alternative due to its potent antioxidant properties, although these properties are highly dependent on the source and extraction method.

**Table 5:** Literature review of the antioxidant activity of lignin

Lignin type	Form	Methodology	Results	Ref
Kraft fractions	Crude	DPPH TPC^a^	Inhibition 62.2–68.2% 26.8–35%	[136]
EHL fractions (corn straw)	Crude	DPPH TPC	IC_50_^b^: 64.76–240.15 µg/mL 155.41–246.13 mg GAE/g	[148]
Kraft fractions (eucalyptus)	Crude	DPPH TPC	IC_50_: 7–10 µg/mL 35–50 mg GAE/g	[149]
Kraft (eucalyptus)	Crude	ABTS DPPH TPC	IC_50_: 3.9 µg/mL IC_50_: 9.4 µg/mL 439.1 mg GAE/g	[139]
Organosolv (corncob)	Crude Nanoparticles	ABTS	IC_50_: 27 µg/mL IC_50_: 88 µg/mL	[38]
Organosolv (walnut shells)	Crude	ABTS DPPH TPC	IC_50_: 9.63 µg/mL IC_50_: 19.17 µg/mL 281.5 mg GAE/g	[122]
Acid–alkali treatment (elephant grass)	Crude Nanoparticles	DPPH	IC_50_: 20 µg/mL IC_50_: 12 µg/mL	[137]
Extraction with NaOH (sugarcane bagasse)	Crude	DPPH	IC_50_: 0.38 µg/mL	[150]
Alkali (cornstalk)	Nanoparticles	DPPH	Inhibition (3 h): 97.9%	[138]
Organosolv (spruce) Kraft (spruce) Organosolv (eucalyptus) Kraft (eucalyptus) Organosolv (spruce) Kraft (spruce) Organosolv (eucalyptus) Kraft (eucalyptus)	Crude	DPPH ABTS	IC_50_: 15.85 µg/mL IC_50_: 16.63 µg/mL IC_50_: 12.85 µg/mL IC_50_: 22.75 µg/mL IC_50_: 3.51 µg/mL IC_50_: 3.47 µg/mL IC_50_: 4.22 µg/mL IC_50_: 5.46 µg/mL	[134]

^a^ TPC analyses were all performed using the Folin-Ciocalteu method, ^b^IC_50_ is half-maximal inhibitory concentration

### UV protection

**Fig. 8:**
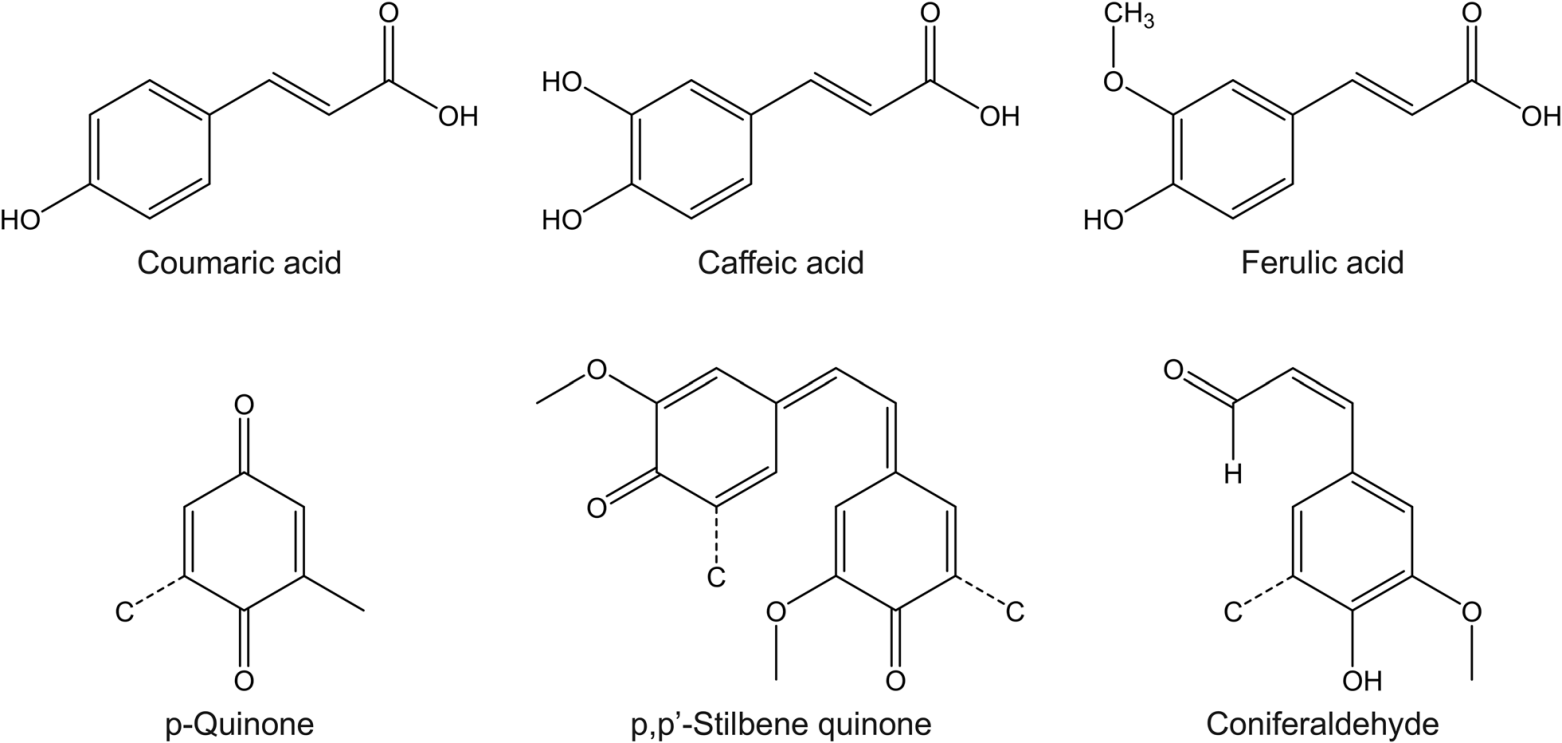
Chromophores found in the lignin structure. Illustration adapted with permission from [151]. Copyright 2021, Royal Society of Chemistry

Lignin is reported to absorb a broad spectrum of UV light, including UVA (320–400 nm) and UVB (290–320 nm) radiations, which are responsible for promoting skin aging, sunburn, and skin cancer [141, 151]. The UV absorption property is attributed to the presence of chromophore groups in the lignin structure (Fig. 8), such as carbonyls, carbon–carbon double bonds, and aromatic groups, as well as auxochrome groups, including hydroxyls and methoxyl groups [152]. Due to this, lignin has been explored as a greener alternative to conventional synthetic sun blockers, which are reported to cause allergic reactions and neurotoxic effects [153]. Common synthetic active ingredients present in sunscreens include organic filters, such as avobenzone, oxybenzone, octocrylene, aminobenzoic acid, and homosalate, as well as inorganic filters, including titanium dioxide and zinc oxide [154, 155].

Several studies evidenced the ability of lignin to act as a sunscreen agent, by increasing the Sun Protection Factor (SPF) of formulations [33, 134, 153, 156, 157]. According to the FDA (U.S. Food and Drug Administration), SPF is the numerical ratio of the UV radiation required to produce sunburn on sunscreen-protected skin divided by the UV radiation required to produce sunburn on unprotected skin [154]. In other words, the SPF value indicates a sunscreen's protection level; for example, an SPF 50 sunscreen provides higher protection than an SPF 15 sunscreen. However, the SPF value indicates only the sunscreen's protection against UVB rays, as tests are performed only for this radiation. In this regard, broad-spectrum tests can be performed to assess the formulation's UVA protection [154]. In the literature, in vitro tests are typically used to measure the SPF of lignin-based sunscreens, in which commercial or in-house base creams or sunscreens containing different loadings of crude lignin or CLPs are evaluated. The test consists of spreading a standard dose (2 mg/cm^2^) of the formulation onto the surface of a transpore tape, followed by a UV transmittance reading in a UV spectrophotometer equipped with a solid sample holder or an integrating sphere [122, 124, 151, 157]. The SPF is usually determined by the following Eq. 4:

4 $$SPF=\sum_{290}^{400}{E}_{\lambda }{S}_{\lambda }/\sum_{290}^{400}{E}_{\lambda }{S}_{\lambda }{T}_{\lambda }$$

where ${E}_{\lambda }$ is the erythemal spectral effectiveness, ${S}_{\lambda }$ is the solar spectral irradiance, and ${T}_{\lambda }$ is the spectral transmittance of the sample [122, 124, 151].

Qian et al. [158] explored the UV absorption of representative samples of technical lignins, namely Kraft, organosolv, soda, lignosulfonate, and enzymatic hydrolysis lignin. They blended each lignin with a moisturizing cream with no UV absorption and detected that lignin increased the SPF of the formulations. More interestingly, when lignins were combined with a sunscreen, they boosted its SPF value more than twice. For example, the moisturizing cream containing 10% organosolv lignin presented an SPF value of 8.66 ± 0.25. In contrast, the sunscreen that initially contained an SPF of 18.22 ± 0.92 increased its value to 91.61 ± 8.47 after the addition of the same 10% organosolv lignin [158]. Other studies have reported the same behavior, suggesting that lignin has a synergistic effect with compounds present in sunscreen [124, 153, 156, 157]. To further explore this, Lee et al. [124] blended enzymatic hydrolysis lignin nanoparticles with sunscreens incorporating organic or inorganic filters. They found that only the sunscreen with organic filters was significantly impacted by the addition of lignin, suggesting that it interacts with aromatic compounds present in the sunscreen, such as octocrylene, octisalate, and ethylhexyl triazone [124].

**Fig. 9:**
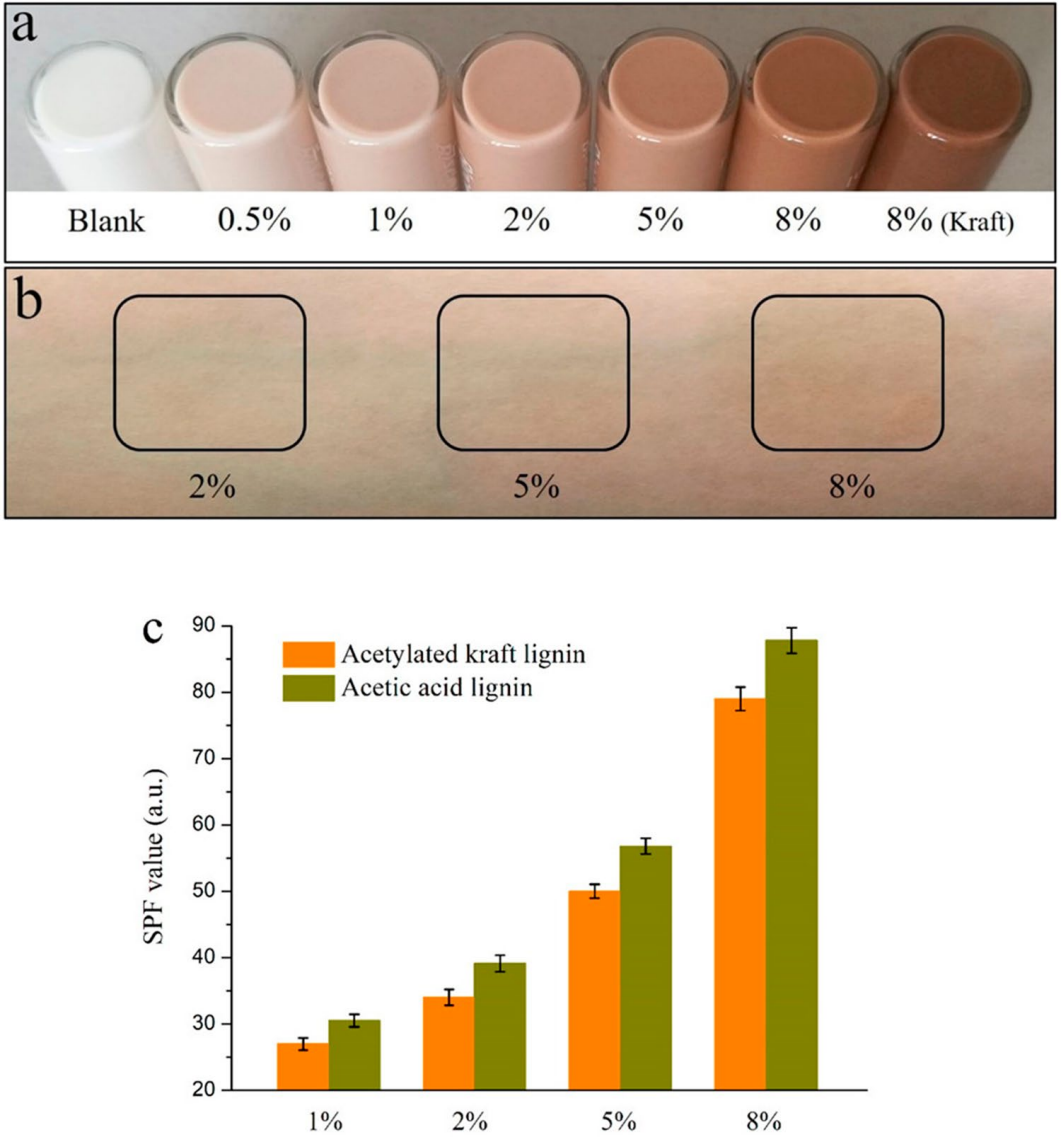
**a** Commercial sunscreen (SFP 15) blended with different dosages of lignin microspheres from bamboo acetic acid or acetylated eucalyptus kraft lignin, **b** skin-staining performances of lignin-based sunscreens, and (c) SPF values of the lignin-based sunscreens. Reprinted with permission from [159]. Copyright 2019, American Chemical Society

Studies have shown that lignin particles have better UV performance than their crude form [33, 123, 124]. Qian et al. [123] prepared lignin particles of varying sizes and incorporated them into a skin cream. Regardless of the particle size, all lignin samples exhibited better UV absorption than crude lignin. Smaller particles showed higher absorption in the UVB range, while larger particles were more effective in the UVA range. This behavior was attributed to the spatial distribution of hydrophilic and hydrophobic groups within the lignin particles. Hydrophilic groups tend to be located on the particle surface, whereas hydrophobic groups are concentrated in the core. Smaller particles, with an average size of 50 nm, possess a larger surface area; thus, their hydrophilic groups could expand the spectrum and enhance the absorbance in the UVB area, while the hydrophobic groups were entrapped inside, weakening the absorption of particles at the UVA region [123]. Furthermore, Zhang et al. [159] showed a significant improvement of the SPF value after incorporating acetic acid lignin microspheres to a commercial lotion originally having SPF 15 (Fig. 9), which achieved an SPF value of 87.2 at the addition amount of 8%.

Table 6 summarizes studies involving crude lignin or CLPs incorporated into creams or sunscreens, along with the corresponding SPF values achieved for each formulation. It is important noting that only studies employing the most accurate in vitro method, specifically those using an integrating sphere for SPF determination, were included. This is due to the fact that many reported SPF values in the literature are based on less reliable techniques. Overall, the results published to date indicate that lignin itself is not sufficient to function as a UV filter capable of covering the entire UV spectrum or achieving the required SPF. However, it can serve effectively as a UV-protective booster, partially replacing synthetic sunscreen actives and thereby reducing their concentration in formulations.

**Table 6:** Literature review on sun protection factor (SPF) of lignin

Lignin type	Form	Vehicle	Lignin concentration	in vitro SPF	Refs.
Lignin extracted from sugarcane bagasse	Crude	Blemish balm cream	5 wt %	9.5	[160]
Enzymatically hydrolyzed lignin (EHL)	Nanoparticles with alkaline treatment	Cream with SPF 1.1 Cream with SPF 19.0 Cream with SPF 55.2	5%	3.6 38.3 87	[161]
EHL	Crude	Pure cream	2.5 wt % 5 wt % 10 wt %	2.9 4.1 5.4	[123]
EHL	Colloidal particles (EHL-S)	Pure cream	2.5 wt % 5 wt % 10 wt %	3.9 6.8 9.1	[123]
Alkali	Crude	Pure cream	0% 5% 10%	1 4 6	[157]
Alkali 370	Crude	Pure cream	0% 5% 10%	1 4 7	[158]
Organosolv	Crude	Pure cream	0% 5% 10%	1 7 9	[158]
Alkali 370	Crude	Cream with SPF 15	0% 5% 10%	18 54 77	[158]
Organosolv	Crude	Cream with SPF 15	0% 5% 10%	18 56 92	[158]

### Lignin safety for human use

**Fig. 10:**
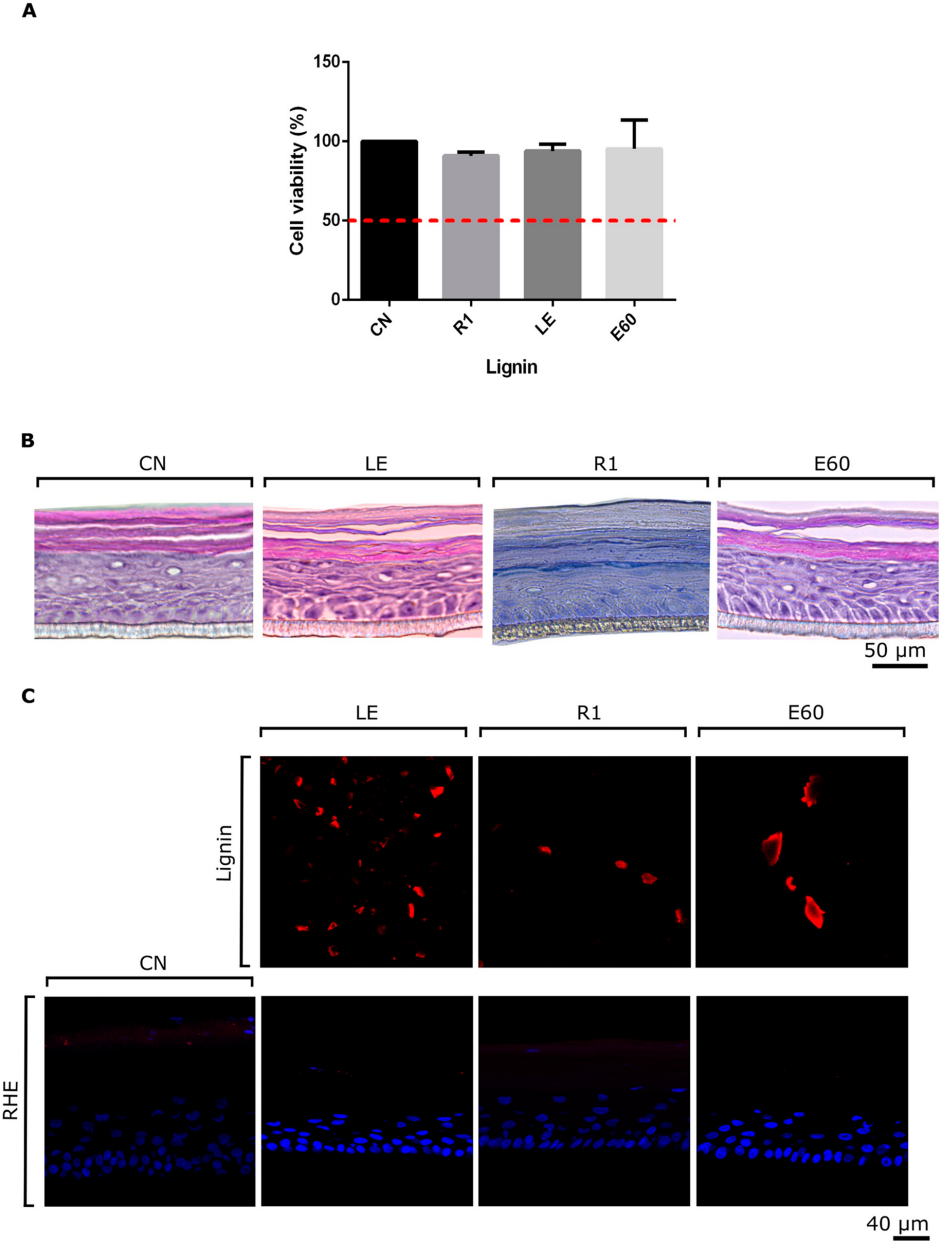
Skin irritation test of kraft lignins (LE, R1 and E60): **a** Cell viability, **b** Morphological evaluation of SkinVitro—RHE after exposure to different lignins, and **c** Evaluation of lignin penetrability in RHEs. Reprinted with permission from [164]. Copyright 2022, Elsevier

Despite lignin’s relevant properties for cosmetics, guaranteeing its safety is of utmost importance in proceeding with its application in human care fields [152]. The toxicity of lignin is commonly evaluated using in vitro cell viability and cell death assays [152, 162]. An increasing number of studies have employed cytotoxicity tests to evaluate lignin safety, with examples summarized in Table 7. Most lignins generally present no or low cytotoxicity, sometimes showing concentration and time dependencies [38, 152]. However, considering the wide range of lignin sources, extraction methodologies, and modifications, it is always necessary to verify the potential toxicity of the specific lignin formulation to validate its use for human application [16]. Recent studies are performing more specific tests for lignin-based cosmetic applications such as the patch test [160, 163]. For example, Hadjiefstathiou et al. [163] performed the patch test to emulsion formulations containing soda lignin (Protobind1000) and reported that both formulations were no irritant after an application for 48 consecutive hours on 12 volunteers. In vitro skin irritation test was performed by Gagosian et al. [164] following the OECD TG 439 guideline with SkinVitro-RHE, an in-house recon structed human epidermis model. The study evaluated kraft lignin and two enzymatically modified derivatives for skin irritation. None of the samples induced irritation or epidermal cell damage, and autofluorescence analysis showed no lignin penetration into the skin model (Fig. 10).

**Table 7:** Literature review on cytotoxic studies of lignin

Lignin	Cell line	Concentration (mg/mL)	Exposure time (h)	Cytotoxicity results	Refs.
Organosolv lignin from hazelnut and walnut shells	Mouse fibroblast 3T3	0.01–1	24, 48, 72	Cell viability lower than 70% after 48 h for 0.5 and 1 mg/mL	[152]
LignoBoost softwood Kraft lignin (nanoparticles)	Human cancer: MDA-MB-231, MCF-7, PC3- MM2, and Caco-2 Non-tumor: KG1 and EA.hy926	25–250	6, 24	Low cytotoxicity at concentrations up to 100 mg/mL	[165]
Organosolv lignin from corncob (nanoparticles)	Human colon adenocarcinoma Caco-2	0.005–0.2	4, 24	Viable cells for all doses after 4 h, and up to 0.1 mg/mL after 24 h	[38]
Alkali lignin and calcium lignosulfonate	Human colon adenocarcinoma Caco-2 and HT-29	0.005–50	24	High cytotoxicity revealed by low values of IC50a of about 4–7 µg/mL	[166]
Kraft lignin (nanoparticles)	Human embryonic kidney HEK-293	0.05–1	24	No cytotoxic effects	[36]
Aquasolv lignin from wheat straw	Human colon adenocarcinoma Caco-2	0–20	4	No cytotoxic effects	[167]
Soda, Lignosulfonate, Curan 100, and Steam Explosion lignins	Human keratinocyte HaCaT and mouse fibroblast 3T3	31.5–1	24, 48, 72	IC50 values of about 0.4 g/L for HaCaT. For 3T3, it was time-dependent, with Curan 100 the most cytotoxic lignin	[142]
Kraft lignin from eucalyptus	Chinese hamster ovary CHO	0.01–1	1	Cell viability up to 0.2 g/L	[139]
Lignin fractions from Acacia nilotica wood	Human hepatic stellate cells HHSteCs	0.0005–0.5	4	IC50: ≥ 100 µg/mL	[168]
Organosolv lignin (nanoparticles)	Human colon adenocarcinoma Caco-2	–	24	No cytotoxic effects	[169]
Organosolv lignin (nanoparticles)	Mouse dendritic DC2.4	0.05–1	24	No cytotoxic effects	[170]
Organosolv lignin (nanoparticles)	Human keratinocyte HaCaT	0.05–0.1	24	No cytotoxic effects	[171]
Alkali lignin (nanoparticles)	Mouse fibroblast 3T3	0–1	72	Cell viability over 90% for concentrations lower than 0.5 g/L	[172]
Calcium lignosulfonate lignin (nanoparticles)	HeLa	0.002–1	4	Low or no cytotoxicity at concentrations up to 1 g/L	[173]
Alkali lignin grafted with poly(N-isopropylacrylamide) (Pickering stabilizers)	Lewis lung carcinoma LLC	–	48	No cytotoxic effects	[114]
Pine Kraft lignin (nanoparticles)	LLC and A549	0.025–0.25	24, 48, 72	Low or no cytotoxicity at concentrations up to 0.25 g/L for 72 h (A549) or 24 h (LLC)	[174]
Alkali lignin grafted with 2–4-benzoyl-3-hydroxyphenyl acrylate (BHA)	Human keratinocyte HaCaT	0–1	24	No cytotoxic effects	[175]
Soda lignin from oil palm empty fruit bunch (nanoparticles)	Human umbilical vein EA.hy926	0.001–51.2	–	No cytotoxic effects	[176]
Enzymatic hydrolysis lignin (nanoparticles)	HeLa	0.025–0.15	48	No cytotoxic effects	[177]
Alkali lignin grafted with polydopamine (nanocapsules)	Human keratinocyte HaCaT	0.1–5	24	No cytotoxic effects	[178]
Lignin from Crataeva tapia leaves	Human PMBC	2.5–80 (× 10–3)	24	No cytotoxic effects	[141]

Some studies have reported a correlation between lignin cytotoxicity and its molecular weight, indicating that lignins with higher molecular weights tend to exhibit lower cytotoxicity. This phenomenon is also associated with the antioxidant activity, where lignins with more potent antioxidant activity, typically those with lower molecular weights, tend to display higher cytotoxic effects [139, 142, 167]. However, the underlying chemical mechanisms driving this effect still need to be elucidated.

The form of lignin also appears to influence its cytotoxicity. Several studies suggest that lignin nanoparticles exhibit lower cytotoxicity compared to their bulk counterparts. For instance, lignin nanoparticles derived from various sources (e.g., Kraft, organosolv, and soda processes) have demonstrated cell viability rates exceeding 70% [165, 171, 176]. Despite these findings, the wide variability in lignin types, as shown in Table 7, complicates the efforts to generalize conclusions about lignin’s cytotoxicity. This variability underscores the need for further systematic research in this area.

### Current market status of lignin for cosmetics

The use of lignin as a functional ingredient in the cosmetic industry is a relatively recent development. Commercialization began in 2021, when the German biotechnology company Lignopure GmbH introduced LignoBase, the first lignin-based ingredient specifically designed for cosmetic applications [128]. LignoBase is produced via particle engineering using spray-drying technology and exhibits multifunctional properties, including antioxidant activity, oil control, natural coloration, and SPF-boosting effects. These characteristics enable its use across a wide range of formulations, including skin care, sun care, makeup, and hair care products [128].

More recently, LignoGuard® SPF Booster, a lignin-based ingredient composed of CLPs, was launched by Lignovations in 2024 [179]. LignoGuard functions as an SPF booster, antioxidant, and emulsion stabilizer, and is intended for use in sunscreens, BB/CC creams, face masks, creams, makeup, and anti-aging formulations [179].

Despite the availability of these commercial lignin-based ingredients, their use remains largely confined to business-to-business (B2B) supply chains. Consequently, consumer-facing cosmetic products that explicitly incorporate lignin remain limited and are predominantly at the formulation or early market introduction stage. Overall, the market for lignin-based cosmetic ingredients is recent but expanding, and broader adoption in finished cosmetic products is expected in the coming years, drive by increasing formulation know-how and rising market awareness.

## Challenges and future perspectives

The multifunctional and sustainable nature of CLPs offers a promising pathway toward a new generation of high-value cosmetic products. CLP-stabilized Pickering emulsions are particularly attractive because they are surfactant-free, reducing the risk of allergic reactions and mitigating the environmental impact associated with conventional synthetic emulsifiers. Beyond stabilization, CLPs can provide added functionalities such as antioxidant activity and UV protection, which may reduce the need for synthetic additives in cosmetic formulations.

Despite these advantages, several critical challenges must be addressed before CLPs can be widely adopted in cosmetic applications. First, while CLPs demonstrate emulsifying performance, systematic comparisons with commercial emulsifiers are still limited. Therefore, more research is needed to assess their stability under relevant cosmetic environment (e.g., under pH and temperature variations) and their compatibility with other formulation ingredients. Further limitation relates to emulsifier dosage since most conventional emulsifiers are effective at relatively low concentrations, whereas CLPs often require higher loadings, which could increase formulation costs.

Additional challenge concerns the functional benefits of CLPs, for example, their photoprotective potential. Although lignin is frequently reported to possess UV-blocking properties, most studies use non-standardized testing methods that do not provide reliable information about SPF values or overall photoprotection. When validated protocols are applied, the observed SPF is typically low, or effective protection requires high CLP concentrations. Future research should therefore focus on developing standardized testing approaches and exploring formulation strategies that optimize CLP-based UV protection.

Safety and regulatory considerations are also critical. The biological effects of CLPs are concentration-dependent, and an optimal balance must be achieved between safety and functionality. Furthermore, the intrinsic brownish color of lignin represents an aesthetic barrier, as the cosmetic industry still largely favors white creams and lotions. Overcoming this challenge will require not only technical advances in particle modification or formulation design but also a shift in consumer perception toward natural and sustainable products.

As future perspectives, progress in this field will benefit from the development of experimental protocols specifically designed to characterize the complex structural and functional properties of lignin in colloidal form. In parallel, the growing involvement of startups and industrial players in commercializing CLPs across different sectors, including cosmetics, will accelerate translation from the laboratory to the market. While significant challenges remain, continued innovation in both science and consumer engagement positions CLPs as a transformative material for the future of sustainable cosmetic formulations.

## Conclusions

The development of CLPs has opened new opportunities for sustainable material innovation in cosmetics. Advances in CLP formation have highlighted how intrinsic lignin parameters and production variables determine their properties and applicability. When applied as stabilizers in Pickering emulsions, CLPs offer an attractive surfactant-free alternative, although emulsion stability is strongly influenced by particle concentration, wettability, and interfacial behavior. Importantly, CLP-stabilized emulsions not only overcome environmental and safety concerns of traditional synthetic emulsifiers but also introduce additional benefits, including antioxidant activity and UV protection, which also reduce reliance on synthetic additives. Further progress will depend on optimizing CLP production methods, establishing reliable protocols for correlating their functional properties, and overcoming application barriers such as the color and dosage requirements. With further research and industry engagement, CLPs hold strong potential to advance both the performance and sustainability of Pickering emulsion products.

## Data Availability

Data sharing is not applicable to this article.
